# Modulation of Oxidative Stress: Pharmaceutical and Pharmacological Aspects

**DOI:** 10.1155/2016/6023417

**Published:** 2016-02-24

**Authors:** Liudmila Korkina, Tomris Ozben, Luciano Saso

**Affiliations:** ^1^Centre of Innovative Biotechnological Investigations (CIBI-Nanolab), 197 Vernadskogo Prospekt, Moscow 119571, Russia; ^2^Department of Biochemistry, Akdeniz University Medical Faculty Campus, Dumlupınar Street, 07070 Antalya, Turkey; ^3^Department of Physiology and Pharmacology “Vittorio Erspamer”, Sapienza University of Rome, Piazzale Aldo Moro 5, 00185 Rome, Italy

Notwithstanding the fact that the multiple roles of oxidative stress in human biology and pathology have been intensely discussed over the last half century, the problem is still far beyond our full comprehension. Thus, in a comparatively short history of oxidative medicine, the roles of two major heroes, free radicals and antioxidants, have been entirely redefined. Free radicals and other reactive oxygen and nitrogen species, widely recognized two-three decades ago as absolute evils leading to and/or accompanying damage to biologically important molecules and structures, have been recently transformed into positive actors, in the appreciation of their essential impact in the intracellular signaling on the organism's defense against biotic and abiotic stresses. Several original research papers published in this special issue have been focused on this subject.

The evidence for detrimental cross interaction of reactive oxygen and nitrogen species was shown in a single clinical case report of rare human disease (Leber's Hereditary Optic Neuropathy (M. Falabella et al.)). The chronic change in the NO homeostasis and enhanced superoxide availability contributed to dysfunction of peripheral blood mononuclear cells derived from a patient due to a cooperative action of nitrosative and oxidative stresses in driving on the genetically determined pathology.

Relations between endothelial nitric oxide synthase genotypes and oxidative stress markers in patients with larynx cancer were studied in an original research (K. Yanar et al.). The results indicated a potential relationship among G894T polymorphism of NOS3 and impaired redox homeostasis that may influence the risk of laryngeal cancer.

An interesting mechanism of adaptation to oxidative stress evolved in enterohemorrhagic* Escherichia coli*, consisting of upregulation of Shiga toxin production by prophages of the bacteria in response to H_2_O_2_ excretion by infected human neutrophils, was described by K. Licznerska and coauthors. The intestinal haemorrhage induced by this type of bacteria depends mainly on Shiga toxin cytotoxicity. This way enterohemorrhagic* Escherichia coli* become tolerant to natural redox-based antibacterial defense of the host organism. One could assume that the modulation of hydrogen peroxide production by human neutrophils could be a promising strategy against this type of bacterial virulence/toxicity. The role of the* exo-xis* region of the bacterial genome in the induction of Shiga toxin-converting prophages is further elucidated by the same group of authors (K. Licznerska and coauthors).

Unfortunately, the great hope that direct antioxidants could be the panacea resolving practically all health problems has vanished, due to the growing number of inconclusive or negative data from epidemiological and clinical studies. The current state of uncertainty regarding feasibility of antioxidant therapy is partly due to pitfalls of biologically relevant and reliable methods of determination of free radical scavenging and antioxidant capacities of isolated substances and compositions. The paper by M. Colmán-Martínez and coauthors suggests a simple and accurate method for simultaneous quantification of carotenes, xanthophylls, and retinol in human plasma based on reversed phase high-performance liquid chromatography coupled with diode array detector (HPLC-DAD).

An innovative approach to modulate and maintain normal redox profile in order to achieve desirable therapeutic effects has recently become a leading one in the management of a variety of redox-dependent pathologies with unmet as yet therapeutic needs. Thus, comprehensive review by V. Chiurchiù and coauthors attempted to answer the question whether modulation of oxidative stress could be therapeutically feasible in the treatment of severe neurodegenerative diseases, such as Alzheimer's and Parkinson's diseases, amyotrophic lateral sclerosis, multiple sclerosis, and hereditary spastic paraplegia. Scrupulously analyzing redox-connected mechanisms underlying the pathogenesis and multiple redox fingerprints characteristic for these diseases, authors came to the conclusion that antioxidants seem to limit the oxidative tissue damage through inhibition of inflammatory events or exerting neuroprotective properties. They also emphasize that the main problem is embodied by the necessity to develop antioxidants that are able to cross the blood-brain barrier. The authors strongly consider that targeted specific redox modulators like dimethyl fumarate rather than conventional dietary antioxidants represent a novel avenue in the management of these human diseases.

Within the same line, the effects of various pharmaceuticals, nutraceuticals, some novel potential pharmacological approaches, and physical exercise on hypercholesterolemia-induced oxidative/nitrative stress and subsequent cardiac dysfunction are discussed in the review (C. Csonka et al.). The authors are focused on 3 different approaches: (i) cholesterol-lowering therapies which attenuate oxidative/nitrosative stress; (ii) combined cholesterol-lowering and antioxidant protocols; and (iii) inducers of endogenous antioxidants or inhibitors of prooxidant enzymes as promising drugs for the prevention and/or treatment of cholesterol-induced cardiac dysfunction. Among pharmaceuticals, statins, Ezetimibe, niacin, fibrates, and an antidiabetic drug Rosiglitazone are discussed in detail. As potent nutraceuticals, antioxidant vitamins, coenzyme Q10, flavonoids (rutin, quercetin, naringin, etc.), green tea catechins, and resveratrol are listed. Novel modulators of miRNAs leading to attenuation of hypercholesterolemia-induced oxidative/nitrosative stress are under development and investigation.

The review article from the Chinese group (Z.-W. Zhang et al.) highlights the potential of mitochondrion-permeable antioxidants in the management of oxidative burst-mediated acute inflammatory conditions. The authors compare and evaluate well-known mitochondrion-permeable antioxidants, such as edaravone, idebenone, alpha-lipoic acid, carotenoids, vitamin E, and coenzyme Q10. In addition, they focus on mitochondria-targeted MitoQ and SkQ antioxidants and propose astaxanthin, a carotenoid, for acute inflammatory conditions characterized by pathologically increased oxidative burst, for example, avian influenza with acute respiratory distress syndrome and ischemia-reperfusion. The limitations of the antioxidant use including adverse health effects under such acute conditions are discussed.

Negative conclusions were drawn (M. Graziani and coauthors) on feasibility of adjuvant therapy of cardiovascular and hepatic toxicity of cocaine with modulators of oxidative stress (N-acetylcysteine, SOD mimetics, nitroxides and nitrones, mitochondria targeting antioxidants, and NADPH oxidase and xanthine oxidase inhibitors,) notwithstanding multiple redox fingerprints in the toxicity of drug abuse. The discrepancy was ascribed to pitfalls in the clinical studies design, to the use of single instead of several targeted oxidative stress modulators, and to the lack of specific, reliable, and reproducible markers of oxidative stress.

An outstanding comprehensive review by N. Sallam and I. Laher concentrates on recent data and modern hypotheses on how physical exercise modulates oxidative stress and inflammation in ageing and cardiovascular diseases. A necessity for strictly individualized programs of age and cardiovascular pathology preventive physical exercises is based on the individual sensitivity to the type, intensity, frequency, and duration of physical challenge. As target organs for protective redox balancing and anti-inflammatory exercises, there are adipose tissue, skeletal muscles, immune system, and cardiovascular system components. The following molecular pathways mediate therapeutic and/or prophylactic effects of physical exercise: redox-sensitive transcription factors, pro- and anti-inflammatory cytokines, antioxidant and prooxidant enzymes, and repair proteins.

Another review (A. Cort et al.) describes in detail a complex redox pattern underlying huge multiple drug resistance (MDR) problem in antitumour therapies as well as the development and clinical use of oxidative stress modulators to combat MDR, thus enhancing efficacy of anticancer protocols. In the authors' opinion, redox active drugs (pro- or antioxidants) targeting an axis consisting of drug transporters, aryl hydrocarbon receptor, phase I/II metabolic enzymes, and the inducible Nrf2-linked pathway could provide a valid and promising way to overcome a dreadful obstacle of MDR in cancer therapies.

Reflecting the general state of the art in the development of novel biologically available antioxidants/redox modulators, a majority of original research papers dedicated to the testing of their pharmacological properties have been presented in the* in vitro* systems or* in vivo* animal experiments. The effects of the clinically used copper chelator D-penicillamine in the catecholamine model of acute myocardial injury were tested in cardiomyoblast cell line H9c2 and in Wistar Han rats (M. Říha et al.). D-Penicillamine had a protective effect against catecholamine-induced injury both* in vitro* and* in vivo*. The classical NOX inhibitors apocynin and diphenyleneiodonium impaired proliferation of cultivated mouse embryonic stem cells (J. Kučera et al.) that clearly reflected essential role(s) of NOX-produced reactive oxygen species as signalling agents for embryonic cell growth and differentiation. Preexposure of UV-B-irradiated human immortalised keratinocyte cell line (HaCaT) to complexed lycopene (A. Ascenso et al.), a skin located antioxidant, led to a shift of the ratio dead : apoptotic : viable subpopulations towards normal values. The authors concluded that the complexed lycopene might have protective or cytotoxic effects in photodamaged and preneoplastic keratinocytes, while allowing other keratinocytes to accelerate repairing mechanisms and remain viable. A broad group of 1,4-dihydropyridine derivatives as long time known compounds with antioxidant potential have been extensively evaluated during last three decades. A. Velena and coauthors attempt to explain the innovative interest in these agents for biomedical applications. Examples of protective and antioxidant properties of 1,4-dihydropyridine derivatives have been provided in a number of recent* in vitro* studies on low density lipoproteins, mitochondria, microsomes, isolated cells, and cell cultures.

As expected, submissions of clinical data were scarce. Only one paper (C. De Luca and coauthors) depicted results of the clinical-laboratory study on the skin antiageing and systemic redox effects of supplementation with the composition of marine collagen peptides and plant-derived skin-targeting antioxidants (coenzyme Q_10_ + grape skin extract + luteolin + selenium). The data obtained clearly show the improvement of skin properties (elasticity, sebum production, biological age, and dermal ultrasound markers) due to enhanced collagen synthesis without risk of oxidative stress that is usually connected with the synthesis. Moreover, hormesis-like action of the supplementation allowed suggesting this mechanism to be responsible for its antiageing and energizing effects.

Due to the limited space of the special issue, we were not able to concentrate on the issues of extreme importance for discovery and development of safe and clinically efficient redox modulators, such as optimization of delivery systems, pharmacokinetics and metabolism, redox activity of metabolites, and biological markers for the assessment of* in vivo* prooxidant/antioxidant action correlating with clinical outcomes.

*Liudmila Korkina* *Liudmila Korkina* *Tomris Ozben* *Tomris Ozben* *Luciano Saso* *Luciano Saso*

